# Proton pump inhibitors for the treatment of laryngopharyngeal reflux disease: A protocol for systematic review and meta-analysis

**DOI:** 10.1097/MD.0000000000023297

**Published:** 2020-12-04

**Authors:** Xiangyi Liu, Ying Jiang, Haiyan Luo, Haolin Liu

**Affiliations:** a Gansu University of Chinese Medicine; b Affiliated Hospital of Gansu University of Chinese Medicine, Lanzhou, Gansu province; c Tianjin University of Traditional Chinese Medicine, Tianjin, China.

**Keywords:** laryngopharyngeal reflux disease, meta-analysis, protocol, proton pump inhibitors

## Abstract

**Background:**

Laryngopharyngeal Reflux disease refers to abnormal reflux of gastric contents through the esophagus into the throat, which irritates and damages the pharyngeal mucosa, and causes corresponding symptoms. Proton Pump Inhibitors are an important class of gastric acid secretion inhibitors after H2 receptor blockers, which can be used clinically to treat peptic ulcer, abnormal gastric acid secretion and other related diseases. The common clinical drugs include omeprazole, lansoprazole, rabeprazole and so on. Clinical practice has shown that Proton Pump Inhibitors have a good therapeutic effect on Laryngopharyngeal Reflux disease, but evidence of evidence-based medicine is lacking. The purpose of this protocol is to systematically evaluate the efficacy and safety of Proton Pump Inhibitors in the treatment of Laryngopharyngeal Reflux disease and to improve the evidence-based basis for the clinical application of Proton Pump Inhibitors in the treatment of Laryngopharyngeal Reflux disease.

**Methods:**

English computer retrieval database (PubMed, Embase, Web of Science, the Cochrane Library) and Chinese computer retrieval database (Wanfang Database, VIP Information Chinese Journal Service Platform, Chinese Biomedical Database) . In addition, Baidu Scholar and Google Scholar were manually searched for randomized controlled clinical studies on the treatment of laryngeal reflux disease with Proton Pump Inhibitors from the establishment of the database to July 2020. Two researchers independently extracted and evaluated the data of the included studies, and meta-analysis was conducted on the included literatures with RevMan5.3 software without language restrictions.

**Results:**

In this study, the efficacy and safety of Proton Pump Inhibitors in the treatment of Laryngopharyngeal Reflux disease are evaluated by the overall response rate, clinical symptom remission rate and other indicators.

**Conclusions:**

This study will provide reliable evidence-based evidence for the clinical application of Proton Pump Inhibitors in the treatment of Laryngopharyngeal Reflux disease.

OSF Registration number: DOI 10.17605 / OSF.IO / NY6SC

## 1 Introduction

Laryngeal reflux disease is 1 of the diseases that physicians and throat doctors most frequently encountered. Laryngopharyngeal Reflux is the abnormal reflux of gastrointestinal contents such as gastric juice and inorganic salts to the upper esophageal sphincter through the esophagus, which stimulates and damages the pharyngeal mucosa and causes a series of symptoms and signs characterized by pain, cough, phlegm, choking cough, pharynx foreign body sensation and hoarseness symptoms.^[1]^ Common causes of reflux include:

(1) Gastric wall barrier structure and dysfunction, leading to reflux of gastric contents; (2) Obstructive sleep apnea hypopnea syndrome is 1 of the causes of Laryngopharyngeal Reflux.^[2]^ The significant increase of negative thoracic pressure during the onset of the disease can cause gastric reflux and slow down the removal of reflux from the esophagus.^[3]^
(2) Poor eating habits, depression and tension^[4]^ can also cause throat reflux.
(3) At present, the clinic mainly relies on drugs and surgical treatment, and at the same time adjusts the patients’ lifestyle and diet structure.^[5]^ Studies have also shown that extracts of some natural ingredients have a good effect on reflux diseases.^[6]^

Proton Pump Inhibitors are commonly used in the clinical empirical treatment of Laryngopharyngeal Reflux diseases. The therapeutic mechanism is to inhibit H+-K+ -ATPase on gastric wall cells, reduce gastric acid secretion, reduce pepsin activity and block the inflammatory response, thereby reducing the direct damage to the throat.^[7]^ However, the effectiveness of Proton Pump Inhibitors in the treatment of Laryngopharyngeal Reflux disease has long been controversial.

At present, although many clinical research results show that Proton Pump Inhibitors are effective in treating Laryngopharyngeal Reflux disease with high remission rate, but there are some adverse reactions, requiring a long treatment cycle. Moreover, The small number of clinical trials and the differences in study design and efficacy have influenced the promotion of this therapy to some extent. Therefore, in this study, we conducted a meta-analysis to investigate the impact of Proton Pump Inhibitors on quality of life and psychological status in patients with Laryngopharyngeal Reflux disease to provide a reliable evidence-based basis for the treatment of Laryngopharyngeal Reflux disease.

## 2 Methods

### 2.1 Protocol register

This protocol of systematic review and meta-analysis has been drafted under the guidance of the preferred reporting items for systematic reviews and meta-analysis protocols. Moreover, it has been registered on the open science framework (OSF) on October 15. (registration number: DOI 10.17605 / OSF.IO / NY6SC).

### 2.2 Ethics

Since this is a protocol with no patient recruitment and personal information collection, the approval of the ethics committee is not required.

### 2.3 Eligibility criteria

#### 2.3.1 Types of studies

We will collect all available randomized controlled trails on Proton Pump Inhibitors for the treatment of Laryngopharyngeal Reflux disease regardless of blinding, publication status, region, but Language will be restricted to Chinese and English.

#### 2.3.2 The research object

Patients who are clearly diagnosed with laryngeal reflux disease according to the evaluation criteria for positivity of reflux symptom index (RSI) and reflux symptom score (RFS): RSI>13 points, RFS > 7 points; Patients who are initially diagnosed with laryngopharyngeal reflux disease according to signs or clinical symptoms of Laryngopharyngeal Reflux disease have no other complications, and the included samples are not limited in nationality, race, age, gender or course of disease.

#### 2.3.3 Intervening measure

Treatment group: Proton Pump Inhibitor. There is no restriction on dosage form, dosage and course of treatment.; Control group: placebo.

### 2.4 Outcome indicator

(1) Main outcome: overall efficiency
(2) Secondary outcome: reflux symptom index (reflux symptom index, RSI), reflux finding score (reflux finding score, RFS), clinical symptom remission rate, pepsin level, incidence of adverse reactions and so on

### 2.5 Exclusion criteria

(1) Study published repeatedly;
(2) Study whose literature is abstract and conference papers, in which the original data cannot be obtained;
(3) Study whose data is incomplete or where there are obvious errors that cannot be handled after contacting the author;
(4) Study with wrong random method;
(5) Study on patients with chronic underlying diseases (asthma, diabetes, hypertension, liver and kidney dysfunction) and pregnant women;
(6) Experimental study with operative treatment intervention.

### 2.6 Search strategy

Chinese search terms such as Proton Pump Inhibitor, omeprazole, Rabeprazole, laryngopharyngeal reflux disease, Larygnphageal Reflux are searched in Chinese databases, including China Knowledge Network, Wanfang Data Knowledge Service Platform, VIP Information Chinese Journal Service Platform Chinese Journal Service Platform, and China Biomedical Database; English search terms such as Proton Pumps Antagonists, Proton Pumps Inhibitors, Laryngopharyngeal Reflux, Gastropharyngeal Reflux are searched in English databases, including PubMed, EMBASE, Web of Science, the Cochrane Library. In addition, Baidu Scholar and Google Scholar were manually searched. The retrieval time was from the establishment of the database to July 2020, and all domestic and foreign literatures on Proton Pump Inhibitors for the treatment of Laryngopharyngeal Reflux diseases were collected. Take PubMed as an example, and the retrieval strategy is shown in Table 1.

**Table 1:** Search strategy in PubMed database.

Number	Search terms
1	Proton pumps inhibitors [Title/Abstract]
2	PPI[Title/Abstract]
3	Proton pumps antagonists [Title/Abstract]
4	Rabeprazole [Title/Abstract]
5	Esomeprazole [Title/Abstract]
6	Pantoprazole [Title/Abstract]
7	Lansoprazole [Title/Abstract]
8	Omeprazole [Title/Abstract]
9	1 OR 2 OR 3 OR 4 OR 5 OR 6 OR 7 OR 8
10	Laryngopharyngeal reflux disease [Title/Abstract]
11	Gastropharyngeal reflux disease [Title/Abstract]
12	Laryngitis [Title/Abstract]
13	9 OR 10 OR 11 OR 12
14	8 And 13

### 2.7 Data screening and extraction

**Figure 1:**
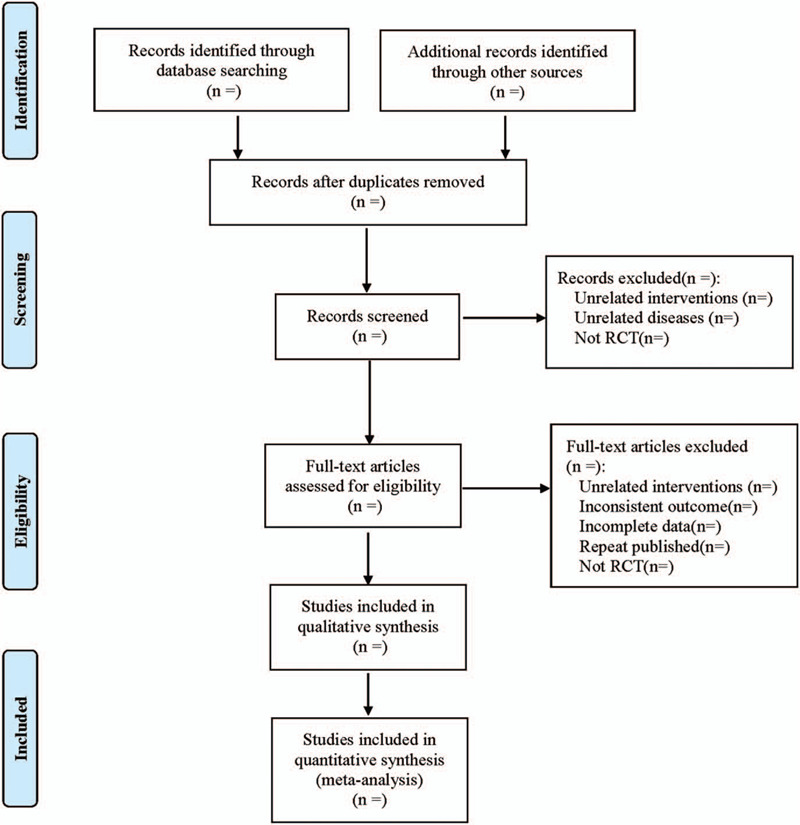
The process of literature filtering.

The 2 researchers made a preliminary screening by reading the title and abstract of the literature, and then made a second screening by reading the full text. Referring to the methods of inclusion in the Cochrane Handbook of Systematic Reviewers (Version 5.0), and according to the preferred reporting items for systematic reviews and meta-analysis flow chart, the literature was selected independently based on the above criteria of inclusion and exclusion, and the results were mutually reviewed. If the results were inconsistent, a third party would participate in the discussion and decision. The extracted literature information includes:

(1) Clinical features (title, first author, publication year and month, sample size, sex ratio, average age, average course of disease);
(2) Intervention measures: the types of Proton Pump Inhibitors used in the treatment group, drug delivery mode, treatment frequency and course of treatment; Treatment measures used in the control group;
(3) Evaluation factors of risk bias in randomized controlled studies;
(4) Observation indicators. The literature screening process is shown in Figure 1.

### 2.8 Literature quality evaluation

The quality of the included literature is evaluated according to the Risk of Bias tool recommended by the Cochrane Handbook. The evaluation items include:

(1) Implementation of blind method,
(2) Generation of random allocation scheme,
(3) Allocation scheme hiding,
(4) Completeness of outcome data,
(5) Whether report the results selectively or not,
(6) Other sources of bias.

According to the results of each study, the above 6 items are evaluated, and the 2 researchers will give low-risk, unclear and high-risk judgments according to the performance of the included literature in the above evaluation items. After each party completes the cross-check, if there is any difference, it needs to be discussed. If no agreement can be reached, it shall be agreed with the researcher of the third party

### 2.9 Statistical analysis

#### 2.9.1 Data analysis and processing

Meta-analysis is performed on the extracted data entry Review Manager 5.3, and the test level (bilateral) equals to0.05. Mean difference (MD) or stand mean difference are used as the effect size of outcome indicators according to the combined situation, and relative ratio is used as the expression of dichotomous variable. Heterogeneity test was performed by χ^2^ and *I*^*2*^ tests, if there is no heterogeneity *(I*^*2*^<50% and *P*>0.1) , the fixed-effect model will be used for analysis; If there is heterogeneity *(I*^*2*^≥50% and *P*≤0.1) , then the random effects model will be used and sensitivity analysis or subgroup analysis will be performed to further exclude the sources of heterogeneity.

#### 2.9.2 Dealing with missing data

If there is missing data in the article, please find an attachment or contact the author via email for additional information. If the author has lost relevant data, the meta-analysis will be abandoned and descriptive analysis will be adopted.

#### 2.9.3 Subgroup analysis

Subgroup analysis is performed according to the treatment group for Proton Pump Inhibitor treatment and placebo; According to the age of the patients, they can be divided into 4 subgroups: minors, young people, middle-aged people and elderly people. Subgroup analysis is performed according to the course of treatment.

#### 2.9.4 Sensitivity analysis

At the same time, the fixed-effect model and the random-effect model are used to carry out meta-analysis on the risk factors for the treatment of Laryngopharyngeal Reflux, and the sensitivity analysis is conducted on the analysis results to exclude the trials with high heterogeneity that may lead to the combined results, and finally the remaining trials are included for analysis.

#### 2.9.5 Assessment of reporting biases

For the major outcome indicators, if the included study was≥10, funnel plots can be used to assess publication bias. If accurate evaluation was required, Egger's and Begg's test were used to quantitatively assess potential publication bias.

#### 2.9.6 Evidence quality evaluation

The Grading of Recommendations Assessment, Development, and Evaluation will be used to assess the quality of evidence. It contains 5 domains (bias risk, consistency, directness, precision, and publication bias). And the quality of evidence will be rated as high, moderate, low, and very low.

## 3 Discussion

Laryngopharyngeal Reflux disease is a common chronic inflammatory disease, due to its lack of specificity, its clinical symptoms are similar to common chronic laryngopharyngeal diseases, which explains why it is easy to be misdiagnosed. At present, RSI and RFS score scales are mostly used to screen patients for diseases, so as to accurately assess the clinical symptoms and signs of patients^[8]^ and quickly and accurately diagnose diseases. Studies have also shown that the changes of pepsin level^[9]^ and gastric bubble size^[10]^ are correlated with Laryngopharyngeal Reflux disease to some extent. After the occurrence of this disease, patients have a relatively obvious sore throat and hoarseness. Some patients also have persistent cough, obvious foreign body sensation in the throat, and even shortness of breath, which seriously affect their life quality. The clinical signs are glottis stenosis, mesangial hyperplasia at the combined site behind the vocal cords, granuloma, diffuse congestion and edema of the vocal cords. If patients are not treated timely and effectively after onset, chronic laryngitis, pharyngitis and laryngeal cancer can easily be caused, which have serious impacts on patients’ physical and mental health.^[11]^ Therefore, rapid diagnosis and effective treatment of Laryngopharyngeal Reflux disease are very important to promote good prognosis and recovery of patients.^[12]^

Gastric wall cells secrete acid through H+-K+ -ATPase on the membrane, which pumps H+ out of the cell in the forms of hydrogen ion and potassium ion exchange. Proton Pump Inhibitors are absorbed into the blood, diffuse into the cells of the gastric wall, and covalently bind to H+-K+ -ATPase, irreversibly inactivating the pump molecules. Gastric acid secretion resumes only when new pump molecules are synthesized and inserted into the cell membrane. Therefore, Proton Pump Inhibitors have strong and persistent effects on gastric acid inhibition, while reducing the secretion of pepsin.^[13]^ This kind of drugs act on the last link of gastric acid secretion, so they can effectively inhibit gastric acid secretion no matter whether there are other factors that stimulate gastric acid secretion^[14]^ or not. However, Proton Pump Inhibitors are not acid resistant and easy to be degraded in an acidic environment. In order to avoid this situation, oral dosage forms are mostly made of capsules, enteric-coated tablets and other preparations. The adverse reactions of this drug mainly include nausea, flatulence, diarrhea, constipation, upper abdominal pain, etc. Rash, Glutamic-pyruvic Transaminase (ALT) and bilirubin elevations also occur, which are generally mild and transient and mostly do not affect treatment.^[15]^ However, about 20% to 30% of patients treated clinically with Proton Pump Inhibitors still develop reflux symptoms.^[16]^ Proton Pump Inhibitors are effective in most patients with Laryngopharyngeal Reflux disease, but are not effective in patients with reflux allergy, weak or non-acid reflux, or neuropsychiatric factors.^[17]^

Currently, there are many widely reported trials of Proton Pump Inhibitors for the treatment of Laryngopharyngeal Reflux disease, but there is a lack of systematic and accurate evaluation. Thus, it is necessary to objectively evaluate the effects of Proton Pump Inhibitors on patients with Laryngopharyngeal Reflux disease through evidence-based medicine, promote the treatment of Proton Pump Inhibitors, and provide scientific and evidence-based western medicine prescriptions for clinical practice. However, this study also has some limitations: The lack of literature in the database and improper retrieval strategies which may lead to missed detection; the lack of high-quality randomized controlled trials with large sample size and rigorous design. At the same time, due to the limitation of language ability, only English and Chinese literature are searched, while studies in other languages may be ignored, which may lead to certain publication bias

## Author contributions

**Data collection**: Xiangyi Liu and Ying Jiang.

**Funding support**: Xiangyi Liu.

**Software operating**: Haiyan Luo.

**Supervision**: Haiyan Luo.

**Literature retrieval**: Haiyan Luo, MMb, Haolin Liu.

**Writing – original draft**: Xiangyi Liu and Ying Jiang.

**Writing – review & editing**: Xiangyi Liu and Haolin Liu.
